# Implementation and evaluation of an interprofessional prescription writing workshop with a simulated electronic prescribing activity for preclerkship medical students

**DOI:** 10.1186/s12909-024-05326-0

**Published:** 2024-04-10

**Authors:** Christopher Guyer, Brittany Stewart, Ziad Khalifa, Linh Pham, Aline H. Saad

**Affiliations:** 1 Clinical Skills Center, School of Medicine, Wayne State University, 320 E Canfield St, Suite 206, Detroit, MI 48201 USA; 2 Department of Pharmacy Practice, Eugene Applebaum College of Pharmacy and Health Sciences, Wayne State University, 259 Mack Ave, Suite 2190, Detroit, MI 48201 USA

**Keywords:** Medical education research, Clinical skills, Interprofessional, Peer-to-peer education

## Abstract

**Background:**

Prescription writing skills are essential for physician practice. This study describes the development and implementation of a curricular intervention focused on improving the knowledge and confidence of preclerkship medical students’ prescription writing practices utilizing an interprofessional education model, with a focus on electronic prescribing.

**Methods:**

Medicine and Pharmacy Faculty from a large, urban university collaborated to develop the content of the workshop and a simulation platform was used for the e-prescribing activity. Second-year medical students attended a mandatory in-person workshop facilitated by fourth-year pharmacy students. A pre and post knowledge test and confidence survey were used to assess students’ knowledge, confidence, and satisfaction. Outcomes from the knowledge test were evaluated with paired-samples proportions tests, and confidence survey data was evaluated with paired t-tests and Wilcoxon signed-rank tests in a pre-post study design.

**Results:**

Students demonstrated a significant increase in prescription writing knowledge and confidence after completing the workshop. On the pre-test, 7% of students (21/284) completed the electronic prescribing assessment correctly and 51% of students (149/295) completed it correctly on the post-test. All items on the confidence survey showed a significant increase in pre- versus post-survey comparisons (*p* < 0.001).

**Conclusions:**

This interprofessional prescription writing workshop facilitated by pharmacy students shows promise for improving the knowledge and confidence of prescription writing and electronic prescribing practices in preclerkship medical students.

**Supplementary Information:**

The online version contains supplementary material available at 10.1186/s12909-024-05326-0.

## Background

Prescription writing is an essential and ubiquitous skill of physician practice [1]. Prescription writing involves integrating diagnostic skills, pharmaceutical knowledge, communication expertise, comprehension of clinical pharmacology principles, and acknowledgement of risk [2]. The Association of American Medical Colleges developed Core Entrustable Professional Activities (EPA) that all medical students are expected to be able to perform when entering residency. They serve as a framework for students and educators to assess real world competencies. EPA 4 focuses on the ability to enter and discuss medication orders and prescriptions [3]. In US medical schools, no standardized prescription writing curriculum or assessment tools exist to facilitate the achievement of EPA 4. The current literature evaluating medical school curriculum interventions aimed at teaching and assessment of prescription writing skills is limited. Previous research has indicated that medical students do not receive sufficient training to demonstrate this important competency [4, 5]. This inadequate training can negatively impact patient safety and quality of life by creating unnecessary medication errors that can result in costly and harmful adverse medical events for patients [6–8]. In fact, medical students do not have independent autonomy to write prescriptions but have opportunities to gain experience in supervised clinical practice. Aronson et al. suggested starting with educational interventions to improve prescribing habits among practicing physicians as the root cause of most errors is due to a lack of a knowledge base related to prescription writing practices [2].

Newby et al. described a pharmacist-led prescribing program for final year medical students in Australia and showed a significant improvement in students’ confidence in prescribing [9]. Various studies supported peer assisted learning and encouraged interprofessional collaboration through facilitating a safe environment for learning and vulnerability [10, 11]. Fourth year pharmacy students receive education throughout the didactic and experiential PharmD curriculum that prepares them with the knowledge and skills needed to deliver prescription writing content to medical students [12]. The Accreditation Council for Pharmacy Education (ACPE) requires didactic content in Pharmacy Law and Regulatory Affairs and many pharmacy schools in the US require a law course in the PharmD curriculum [13]. Introducing prescription writing skills in the preclerkship phase allows medical students to longitudinally reinforce these skills through early clinical experiences (such as in continuity clinics), clerkships, elective rotations, and in residency training.

To decrease medication errors, electronic prescribing (e-prescribing) has been widely adopted and is becoming the standard of practice for prescription writing in the United States to enhance safety and quality [14]. E-prescribing allows the prescriber to send an error-free and legible prescription directly to the pharmacy, reduces potential for prescribing errors, decreases the time required to write a prescription [14] and is an important factor in improving quality patient care [15]. Many states in the US have mandated e-prescribing regulations while others are planning to implement mandates in the future [16], thus a need exists to develop educational activities to teach medical students traditional prescribing best practices and embrace new technology to prepare the next generation of physicians for practice.

A recent study suggested that an interprofessional education model focused on pharmacy students educating medical students about traditional prescription writing practices was effective in improving their confidence in prescription writing [10]. Our study differs from Allen et al. in that its design includes the use of a simulated electronic health record (EHR) and an e-prescribing activity. This study aims to evaluate the effectiveness of an interprofessional prescription writing workshop using a simulated EHR to improve medical students’ knowledge of and confidence with prescription writing practices.

## Methods

### Development of workshop content

During the 2022–2023 academic year at a large single campus urban university, a core group of medicine and pharmacy faculty (one physician and two pharmacists) and fourth year pharmacy students collaborated to develop content for the prescription writing workshop. Learning goals and objectives were developed by pharmacy and medical school faculty with input from the respective curriculum committees based on a needs assessment of the existing preclerkship medical curriculum. Faculty considered foundational concepts for safe prescription writing practices in the workshop design. The workshop content included identifying required elements of a prescription, legal requirements of prescribing, routes of administration, accepted abbreviations, weight-based dosing, common sources of error, and patient counseling.

### Workshop delivery

Medical students participating in their second-year clinical skills course, and fourth year pharmacy students completing their community advanced pharmacy practice experience were recruited to participate in the prescription writing workshop. The program consisted of a self-study component and an in-person workshop facilitated by fourth year pharmacy students. Prior to viewing the self-study materials, medical students completed a baseline 11-question knowledge pretest (Appendix 1) to assess understanding of key definitions and prescription writing concepts and an 11-item confidence survey with a 5-point Likert-scale (1 = strongly disagree, 5 = strongly agree) (Appendix 2). Included in the knowledge pretest, they completed a case-based e-prescribing activity to assess for its entirety and errors. Prior to the live workshop, medical students completed a 30-minute self-study module delivered through Canvas, a web-based learning management system (Instructure, Salt Lake City). The self-study didactic content focused on prescription writing legal/regulatory requirements and was delivered through two pre-recorded videos.

Following the self-study, the in-person 60-minute interprofessional workshops were conducted over two days with groups of ten medical students assigned to two pharmacy students in each room. The pharmacy students facilitated, provided guidance and feedback during four active learning exercises included (1) writing out five prescriptions with all the required elements of a prescription, (2) pediatric weight-based dosing calculations, (3) a simulated electronic prescribing activity through an educational EHR platform, and (4) patient counseling techniques. Activities 3 and 4 were both based upon a prescription for an insulin pen device and pen needles.

At the conclusion of the workshop, medical students completed the same knowledge test and confidence survey as prior to the workshop. To ensure consistency in workshop content delivery, the pharmacy students developed a facilitators’ guide with an answer key and facilitator slides to be presented during the workshop. In preparation for the workshop, pharmacy faculty met with the pharmacy students during three sessions to review the instructional materials and provide training and tips about delivery of the active learning exercises to the medical students. Pharmacy and medical school faculty were present at the workshop for observations and support.

### Assessments and evaluation

During the workshop, students completed formative self-assessment exercises of handwritten prescriptions and electronic prescriptions using a simulated EHR and order entry system. Summative knowledge was assessed through a knowledge-based pre- and post-test. The knowledge test questions assessed the learning objectives including understanding prescription writing legal and regulatory requirements, and best practices to ensure clear written communication between prescribers and pharmacists. Additionally, student confidence was measured prior to the workshop, and confidence and satisfaction were measured within 2 weeks following the completion of the workshop. Confidence questions were derived from the learning objectives to determine students’ perception of their own knowledge before and after completing the self-directed module and the in-person workshop. The post-survey also included questions assessing students’ overall satisfaction with the activities and allowed for narrative evaluation of the course. Although not validated, these assessment tools were piloted prior to their adoption by the pharmacy students who helped in the design of the workshop.

### Data analysis

Microsoft Excel was used to organize the data collected from the knowledge test and the confidence survey. IBM SPSS Statistics (Version 28) and SAS/STAT (version 9.4) were used for statistical analysis. The knowledge pre-test and post-test comparisons were conducted with paired-samples proportions tests due to the binary nature of the variables (correct vs. incorrect). Grading for the simulated e-prescribing activity was performed by medicine and pharmacy faculty, and a senior pharmacy student. All required legal elements of a prescription needed to be present for the assessment to be considered correct. Paired t-tests and Wilcoxon signed-rank tests were used for the Likert survey data to evaluate changes in the responses in the pre and post setting. Only students who completed both pre and post knowledge tests and confidence surveys were included. A standard p-value < 0.05 indicated statistical significance. Individual student pre-tests and post-tests were paired for completion of data analysis. Statistical analysis was performed without adjustment for repeated testing. This study was provided an exempt status by the university’s Institutional Review Board.

## Results

All 298 medical students in the second-year clinical skills course participated in the interprofessional prescription writing workshop. Table 1 details the demographics of the medical students who participated in the workshop.

**Table 1:** Medical student participant demographics

Class size	*N* = 298
Self-identified gender	
Female	152 (51%)
Male	146 (49%)
Mean age (range)	25 (22–40)
Self-identified race	
	115 (39%)
Asian	81 (27%)
Hispanic	35 (12%)
African American	31 (10%)
Other	28 (9%)
Not answered	8 (3%)

### Knowledge-based assessment

Prior to the workshop, 95% (284) of students completed the pre-test, and following the workshop 99% (295) completed the post-test. The average score of the pre-test was 52% and the post-test was 79%. There was a statistically significant improvement in students’ performance on 9 out of 11 of the test questions, as seen in Table 2. Prior to the workshop, 7% (21/284) of students completed the simulated e-prescribing assessment correctly (being complete and having no errors that would require clarification by a pharmacist prior to filling the prescription) while 51% (149/295) completed it correctly following the workshop.

**Table 2:** Pre/post workshop knowledge test scores

Question topic	Pre-test Percent Correct (*N* = 284)	Post-test Percent Correct (*N* = 295)	P-value
Regulatory requirements	25 (9%)	102 (34%)	< 0.001
Prescription elements	190 (67%)	284 (97%)	< 0.001
Electronic prescribing requirements	237 (83%)	286 (97%)	< 0.001
Legal requirements for prescribing controlled substances	265 (93%)	287 (98%)	0.036
Weight-based dosing	193 (68%)	286 (97%)	< 0.001
Acceptable abbreviations	105 (37%)	219 (74%)	< 0.001
Requirements for refills	94 (33%)	215 (73%)	< 0.001
Controlled prescription requirements	275 (97%)	281 (95%)	0.31
Dispense as written	158 (56%)	244 (83%)	< 0.001
Prescription duration and expiration	52 (18%)	214 (72%)	< 0.001
E-prescribing simulation	21 (7%)	149 (51%)	< 0.001

### Confidence and satisfaction assessment

**Fig. 1:**
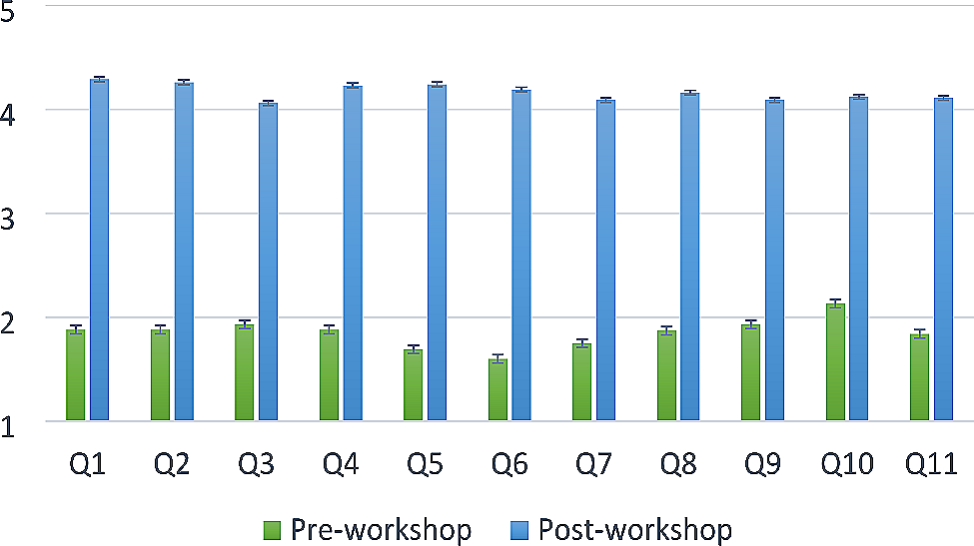
Pre/post workshop confidence survey responses. Average score based on 5-point Likert scale

Of the 298 students who participated in the workshop, 89% (265) completed both the pre- and post-workshop confidence survey. All items on the survey showed a significant increase in pre- and post-survey comparisons with a cumulative average for all 11 items of 1.85 pre-workshop to 4.17 post workshop, (*p* < 0.001). A summary of the pre- and post-workshop confidence survey responses are shown graphically in Fig. 1 and numerically in Table 3. Students somewhat or strongly agreed that the prescription writing workshop: (1) met their expectations, 88% (232/265); (2) added to their knowledge and skills in writing prescriptions that are free of errors, 92% (243/265); (3) that the pharmacy student facilitators were knowledgeable, 98% (261/265); and (4) that the senior pharmacy student facilitators were professional, 98% (261/265). Students’ feedback from open-ended questions included the need for more time in the workshop (*n* = 49), that they enjoyed the sessions (*n* = 10), and that they would benefit from more detailed explanations in the workshop (*n* = 4).

**Table 3:** Pre/post workshop confidence survey responses

Variable	N	Mean	Std Dev	Paired t-test p-value	Wilcoxon signed-rank p-value	Cohen’s_d
Pre Q1	283	1.87	1.08	.	.	.
Pre Q2	283	1.86	1.07	.	.	.
Pre Q3	283	1.93	1.10	.	.	.
Pre Q4	283	1.88	1.10	.	.	.
Pre Q5	282	1.68	0.99	.	.	.
Pre Q6	282	1.59	0.91	.	.	.
Pre Q7	281	1.74	1.00	.	.	.
Pre Q8	281	1.86	1.10	.	.	.
Pre Q9	282	1.92	1.07	.	.	.
Pre Q10	283	2.13	1.16	.	.	.
Pre Q11	283	1.84	1.09	.	.	.
Post Q1	270	4.29	0.79	.	.	.
Post Q2	269	4.26	0.83	.	.	.
Post Q3	269	4.07	0.94	.	.	.
Post Q4	270	4.23	0.86	.	.	.
Post Q5	270	4.24	0.84	.	.	.
Post Q6	268	4.19	0.85	.	.	.
Post Q7	269	4.09	0.92	.	.	.
Post Q8	269	4.16	0.85	.	.	.
Post Q9	267	4.10	0.91	.	.	.
Post Q10	268	4.12	0.87	.	.	.
Post Q11	267	4.12	0.91	.	.	.
Change Q1	269	2.42	1.28	< 0.0001	< 0.0001	1.88
Change Q2	268	2.38	1.38	< 0.0001	< 0.0001	1.72
Change Q3	268	2.13	1.46	< 0.0001	< 0.0001	1.46
Change Q4	269	2.35	1.44	< 0.0001	< 0.0001	1.63
Change Q5	268	2.55	1.32	< 0.0001	< 0.0001	1.93
Change Q6	266	2.59	1.29	< 0.0001	< 0.0001	2.00
Change Q7	266	2.33	1.37	< 0.0001	< 0.0001	1.71
Change Q8	266	2.29	1.38	< 0.0001	< 0.0001	1.66
Change Q9	265	2.16	1.39	< 0.0001	< 0.0001	1.55
Change Q10	267	2.00	1.48	< 0.0001	< 0.0001	1.35
Change Q11	266	2.27	1.38	< 0.0001	< 0.0001	1.65

## Discussion

Medical students’ prescription writing knowledge and confidence improved following completion of an interprofessional prescription writing workshop facilitated by pharmacy students, similar to findings reported by Allen et al. [10]. Unique to this study, there was significant improvement in medical students’ performance on knowledge-based assessment and the simulated e-prescribing activity. The era of e-prescribing standards presents a pivotal time to implement educational strategies to optimize health information technology. To our knowledge there are limited studies investigating e-prescribing educational interventions in undergraduate medical education. Our study showed that medical students could benefit from simulated e-prescribing activities to improve their knowledge and confidence.

Medical students’ knowledge increased significantly in the areas related to required elements for prescription writing, legal requirements, weight-based dosing strategies and approved abbreviations after an interprofessional prescription writing workshop. A 7-fold increase was demonstrated in the number of students correctly completing the simulated e-prescribing assessment. This highlights that active learning can focus teaching strategies on the most important information presented during the workshop to allow the medical students to process and retain what they learned for successful completion of the post-assessment [17]. One of the challenges in creating a consistent educational strategy is the variability in e-prescribing platforms used across different health systems and that the learning environment should reflect clinical practice [18]. The simulation e-prescribing platform used in this study was developed at the university’s medical school with the intent to reflect features found in commonly used e-prescribing platforms. Optimal prescription writing educational training for medical students may need to be a hybrid of interventions including self-study, handwritten activities, group case-based learning, and e-prescribing simulations.

One topic area of struggle for the medical students on the pre- and post-test, although still showing a significant knowledge increase, was related to regulatory requirements in prescription writing. This could be attributed to the fact that this was not a focus of the active learning components during the in-person prescription writing workshop and that medical students don’t receive this in their didactic curriculum. This is an area of opportunity to focus on in the future renditions of the prescription writing workshop and to include more didactic material in the medical school curriculum.

Students’ perceived confidence in their ability to write an electronic prescription dramatically improved after participating in the prescription writing workshop. Following the workshop, medical students’ confidence increased significantly in their ability to identify the legal requirements of a prescription, calculate weight-based dosing, compose electronic prescriptions, provide appropriate patient counseling, and write error-free prescriptions that can be filled by a pharmacist without further clarification. This demonstrates that the workshop facilitation and teaching strategies led by pharmacy students were successful and the medical students and pharmacy students were receptive to the interprofessional peer-to-peer educational learning environment. This is foundational in building interprofessional team collaborations in practice between pharmacists and physicians. A pilot study conducted by Newby et al. showed similar findings that a prescribing program led by pharmacists resulted in significant improvement in medical students’ confidence in prescribing and provided an interprofessional approach to educational prescribing approaches [9].

Exposing medical students to key prescription writing practice concepts during the preclerkship phase of training was conducted with the expectation that skills will be used in clinical rotations to prepare for actual practice, setting a foundation to hone these skills through scaffold learning and ultimately meeting EPA 4 milestone. Additionally, interprofessional education materialized as medical students learned *from* pharmacy students, *about* the practice of pharmacy, and *about* the best practices to provide error-free prescriptions which ultimately impact patient safety. This learning occurred as an active and cooperative process with both medical and pharmacy students bringing their own unique perspectives and experiences to shape and understand their roles and responsibilities as healthcare providers in safe prescription writing practices. This is aligned with a progressive shift toward interprofessional education and team-based patient care [19]. Previous research has suggested that medical students would benefit from learning from pharmacists to better understand the prescribing process [2, 9, 19]. Future reiterations of this workshop will focus on enhancing the learning’s reciprocity and enriching the interprofessional experience by adding an activity where pharmacy students learn from their medical colleagues.

Future research interests include expanding this activity to include other prescribers’ professions (e.g., nurse practitioner, dentistry, and physician assistant programs), examining best practices in learning theories in preclerkship clinical skills teaching, longitudinal assessment of students’ prescription writing skills and overall development of this EPA competency in clerkship, and evaluating students’ perceptions of the interprofessional peer to peer education.

Limitations observed in this study included that the workshop content, knowledge-based test questions, and confidence survey were developed by the program faculty coordinators and not validated prior to being implemented for use. Medical students may have had preexisting knowledge of prescription writing practices which was not assessed as part of this study. This study also lacked a control arm. The pairs of pharmacy student facilitators may have conducted the live workshops with variability among content delivery. Additionally, the workshop and its associated knowledge and confidence assessments were only conducted once over one year at a single institution and long-term retention of prescription writing knowledge is unknown. Opportunities exist for validation of knowledge test questions and assessment of student confidence. Future study is also needed to examine students’ long-term retention of knowledge.

## Conclusions

This study highlights that an interprofessional prescription writing workshop facilitated by pharmacy students positively impacts medical students’ prescription writing knowledge, e-prescribing skills, and confidence.

### Take home messages

- Preclerkship medical students would benefit from curriculum aimed at teaching basic tenets of prescription writing to prepare them for clinical practice.
- Current clinical practice requires prescribers to be familiar with both legacy handwritten prescriptions and e-prescribing.
- Medical students can successfully learn prescription writing skills from pharmacy students in an interprofessional educational activity.
- Students indicated high satisfaction with an interprofessional education activity to learn best practices in prescription writing.

### Electronic supplementary material

Below is the link to the electronic supplementary material.

Supplementary Material 1

Supplementary Material 2

## Data Availability

The datasets used and/or analyzed during the current study are available from the corresponding author on reasonable request.

## Abbreviations

EPA: Entrustable Professional Activities
E-prescribing: Electronic prescribing
EHR: Electronic health record
