# Supplementary material for: Implementation and evaluation of an interprofessional prescription writing workshop with a simulated electronic prescribing activity for preclerkship medical students
Source: BMC Med Educ. 2024 Apr 10;24:394. doi: 10.1186/s12909-024-05326-0 (PMC11005250; doi:10.1186/s12909-024-05326-0)
Supplement: Supplementary file 1 — Supplementary Material 1 [file 12909_2024_5326_MOESM1_ESM.docx]

Appendix 1: Prescription Writing Knowledge Pre/Post Test*

1. Which of the following products would require a diagnosis code on the prescription?
   1. Albuterol HFA inhaler for asthma
   2. Contour Next test strips for diabetes
   3. Lantus Solostar pen for diabetes
   4. Lexapro 20 mg for depression
2. Which of the following is NOT a requirement on a noncontrolled prescription?
   1. DEA number
   2. Instructions (SIG)
   3. Date
   4. Refills
3. There is a legal requirement in Michigan that by 2023 all prescriptions must be transmitted electronically.
   1. True
   2. False
4. Michigan law requires prescribers to run a MAPS report when prescribing controlled substances for greater than a 3-day supply.
   1. True
   2. False
5. Which of the following patient populations is primarily weight-based dosing used for?
   1. Adults
   2. Pediatrics
   3. Older adults
   4. All patients
6. The following abbreviations are approved for use when writing prescription instructions: QD, BID, and TID.
   1. True
   2. False
7. Which statement is correct regarding refills on prescriptions?
   1. C-2 prescriptions can have up to 2 refills
   2. C3-5 prescriptions can have up to 5 refills
   3. Number of refills does not have to be on a noncontrolled prescription
   4. Noncontrolled prescriptions can have an unlimited number of refills
8. All of the following are required elements of a controlled prescription: drug, dose, strength, written quantity, and day supply
   1. True
   2. False
9. DAW1 on a prescription means that the patient prefers a brand name medication.
   1. True
   2. False
10. Which statement is correct regarding expiration dates on prescriptions?
    1. All noncontrolled and controlled prescriptions expire within a year
    2. All controlled prescriptions expire within 6 months
    3. All noncontrolled prescriptions expire within 6 months
    4. C-2 prescriptions expire in 90 days
11. Purvi Patel is a patient you are caring for in your continuity clinic. You have recently diagnosed her with essential hypertension. Her physical exam is unremarkable and a recent basic metabolic profile is within normal limits. You have already counseled her on lifestyle modifications and she has tried them for 6 months without success. Her current blood pressure is less than 20/10 mmHg above goal. You want to start her on monotherapy treatment with chlorthalidone. Navigate to the patient's electronic health record (EHR) below and use it to write the patient a prescription. Use the Micromedex link in the EHR to determine dosing. Upload the pdf of your prescription.
12. Diego Barnes is a pediatric patient you are caring for in your continuity clinic. You have recently diagnosed him with community acquired pneumonia. You want to start him on a course of azithromycin. Navigate to the patient's electronic health record (EHR) below and use it to write the patient a prescription. Use the Micromedex link in the EHR to determine dosing. Upload the pdf of your prescription.

* Questions 1-11 are repeated on both the pre and post workshop knowledge tests. Question 12 is only included on the post-workshop knowledge test.
