# Supplementary material for: Implementation and evaluation of an interprofessional prescription writing workshop with a simulated electronic prescribing activity for preclerkship medical students
Source: BMC Med Educ. 2024 Apr 10;24:394. doi: 10.1186/s12909-024-05326-0 (PMC11005250; doi:10.1186/s12909-024-05326-0)
Supplement: Supplementary file 2 — Supplementary Material 2 [file 12909_2024_5326_MOESM2_ESM.docx]

Appendix 2: Confidence Surveys

| **Confidence Survey Questions Before Attending the Interprofessional Workshop** |
| --- |
| *Before completing the P4/M2 Prescription Writing Workshop, I felt confident in my ability to*:^a^ |
| Identify the required elements of a prescription. |
| Write a prescription using the appropriate routes of administration of dispensed medications. |
| Write a prescription that is free of medical abbreviation errors. |
| Calculate appropriate dose for a weight-based medication. |
| Write a prescription (for a non-controlled medication) that contains all legally required elements. |
| Write a prescription (for a controlled medication) that contains all legally required elements. |
| Write an electronic prescription free of errors. |
| Write a prescription for a medication that has detailed administration instructions. |
| Identify sources of error in prescription writing. |
| Provide appropriate patient counseling for a new prescription. |
| Write a prescription that can be filled by a pharmacist without further clarification. |

^a^Scale for this question was: strongly agree, somewhat agree, neither agree nor disagree, somewhat disagree, strongly disagree

P4= Fourth-Year Student Pharmacist

M2= Second-Year Medical Student

| **Confidence Survey Questions After Attending the Interprofessional Workshop** |
| --- |
| *After completing the P4/M2 Prescription Writing Workshop, I felt confident in my ability to*:^a^ |
| Identify the required elements of a prescription. |
| Write a prescription using the appropriate routes of administration of dispensed medications. |
| Write a prescription that is free of medical abbreviation errors. |
| Calculate appropriate dose for a weight-based medication. |
| Write a prescription (for a non-controlled medication) that contains all legally required elements. |
| Write a prescription (for a controlled medication) that contains all legally required elements. |
| Write an electronic prescription free of errors. |
| Write a prescription for a medication that has detailed administration instructions. |
| Identify sources of error in prescription writing. |
| Provide appropriate patient counseling for a new prescription. |
| Write a prescription that can be filled by a pharmacist without further clarification. |

^a^Scale for this question was: strongly agree, somewhat agree, neither agree nor disagree, somewhat disagree, strongly disagree

P4= Fourth-Year Student Pharmacist

M2= Second-Year Medical Student

| **Additional Questions Following the Interprofessional Workshop^a^** |
| --- |
| Overall, the P4/M2 Prescription Writing Workshop met my expectations. |
| The P4/M2 Prescription Writing Workshop added to my knowledge and skills in writing prescriptions that are free of errors. |
| P4 students were knowledgeable. |
| P4 students were professional. |
| Please share any suggestions you have for improving this workshop. |

^a^Scale for this question was: strongly agree, somewhat agree, neither agree nor disagree, somewhat disagree, strongly disagree

P4= Fourth-Year Student Pharmacist

M2= Second-Year Medical Student
